# A Phenomenological Study of the Lived Illness Experience of Adolescents with Chronic Glomerular Disease

**DOI:** 10.3390/children12121671

**Published:** 2025-12-09

**Authors:** Sug Young Lee

**Affiliations:** Nursing Department, Kunsan College of Nursing, Dong‑Gaejeong‑gil, Gaejeong‑dong, Gunsan-si 54068, Jeollabuk‑do, Republic of Korea; sara27@daum.net; Tel.: +82-63-450-3831

**Keywords:** chronic glomerular disease, adolescent, nephrotic syndrome, resilience, lived experience

## Abstract

**Aim:** This qualitative study explored the fundamental characteristics of the illness experiences among adolescents living with chronic glomerular disease. **Methods**: A phenomenological research approach was employed. In-depth interviews were conducted between May and December 2015 with 13 adolescents aged 14–19 years who were diagnosed with chronic glomerular conditions requiring long-term monitoring and management. Results: Seven thematic clusters emerged from the data: “appearance and worsening of kidney disease symptoms,” “restrictions in daily living,” “unstable self-control,” “changes in relationships with friends,” “sensitivity about a decrease in achievements due to disease,” “efforts to maintain a normal daily life,” and “psychological, physical, and social strengthening.” The core experience was characterized as “overcoming limitations due to chronic disease and demonstrating resilient growth.” **Conclusions**: This study offers valuable insights into how adolescents interpret, cope with, and adapt to chronic renal conditions in their daily lives. The findings underscore the need for developmentally appropriate, interdisciplinary care strategies that include structured psychosocial and educational support to promote resilience and improve quality of life in this population.

## 1. Introduction

Chronic disease refers to a condition requiring ongoing medical care for at least six months and typically characterized by repeated cycles of relapse and remission [1]. Chronic kidney disease (CKD) in children and adolescents is defined as kidney damage or a decline in kidney function that persists for at least three months [2]. However, in pediatric practice, a large proportion of young patients present with recurrent or chronic glomerular disorders—such as nephrotic syndrome, glomerulonephritis, or IgA nephropathy—that may not strictly meet eGFR-based criteria but still require continuous treatment, lifestyle regulation, and psychosocial adaptation. For this reason, these conditions are clinically regarded as part of the broader CKD spectrum in childhood and adolescence.

Recent national data from Korea (2020) report the prevalence of pediatric CKD as approximately 3.7 per million children, with 2.6 per million requiring dialysis [3]. The most common etiologies include glomerulonephritis (41%), chronic pyelonephritis (25%), and congenital or hereditary kidney disorders. Similar trends are observed internationally, where improved medical therapies have significantly enhanced survival and health outcomes in pediatric CKD over the past decade [4]. Yet, despite these medical advances, the psychosocial dimensions of chronic kidney disease—especially in adolescents—remain insufficiently explored.

Adolescents with chronic glomerular disease experience not only physical symptoms such as edema, fatigue, and appetite loss but also profound psychological and social challenges. They frequently report anxiety, depression, and lowered self-esteem [5,6]. Recurrent hospital visits, long-term medication use, and dietary restrictions disrupt school attendance and peer relationships, leading to social withdrawal and academic difficulties [7,8,9]. These burdens are particularly salient during adolescence, a developmental period marked by identity formation, growing autonomy, and peer integration [10]. As adolescents strive to establish independence and future goals, chronic illness can complicate their self-concept and emotional adjustment.

While some studies have addressed chronic illness in pediatric populations, qualitative research focusing specifically on the lived experiences of adolescents with chronic or relapsing glomerular disease remains limited, especially in non-Western contexts. Existing studies often generalize findings across all pediatric CKD cases, overlooking the unique psychosocial realities of adolescents who live with chronic glomerular disorders that persist into early adulthood. For many, these illnesses begin in childhood and continue through adolescence, shaping their developmental trajectory and identity.

Understanding how adolescents make sense of their illness, cope with daily challenges, and maintain psychological resilience is crucial for developing holistic, developmentally appropriate interventions. Accordingly, this study aims to provide an in-depth exploration of the lived experiences of adolescents with chronic glomerular disease. By examining how they perceive, manage, and adapt to their condition within the contexts of family, school, and peer relationships, this research seeks to inform interdisciplinary and age-sensitive strategies for supporting adolescents with chronic kidney-related conditions.

Research Question:

What is the nature of the illness experiences of adolescents with chronic glomerular disease?

## 2. Methods

### 2.1. Study Design

This study employed the phenomenological research approach developed by Colaizzi [9]. Colaizzi’s method emphasizes the identification of common features among all participants rather than focusing on individual differences. This approach was deemed appropriate for exploring the shared, lived experiences of adolescents with chronic or recurrent glomerular disease.

### 2.2. Ethical Considerations

This study received approval from the Institutional Review Board of Dankook University Hospital (Approval No. DKUH2015-04-015, dated 15 April 2015). Before data collection, researchers introduced themselves, explained the study objectives and procedures in detail, and obtained written informed consent from all participants.

For participants under the age of 18, written informed consent was additionally obtained from their legal guardians, in accordance with Korean ethical guidelines for research involving minors. The assent and consent procedures were reviewed and approved by the IRB to ensure compliance with local regulations. Participants were clearly informed that they could withdraw from the study at any time without any impact on their clinical care, and that their privacy and anonymity would be fully protected.

Each participant was assigned a unique identification code. Documents containing personal information were stored separately from interview transcripts. All research materials were kept in password-protected secure storage and were scheduled for permanent destruction following completion of data analysis and publication.

### 2.3. Participant Selection and Data Collection

Participants were adolescents aged between 14 and 19 years who had been diagnosed with chronic or recurrent glomerular diseases (including nephrotic syndrome, glomerulonephritis, IgA nephropathy, nephritis, and asymptomatic hematuria). While the specific diagnoses varied, all required long-term medical monitoring, symptom management, and lifestyle adjustment. Therefore, they were included as part of the broader category of chronic kidney-related conditions in adolescence.

For the purposes of this study, adolescents diagnosed with relapsing or chronic glomerular conditions requiring continuous clinical management for at least six months were considered within the chronic kidney disease (CKD) spectrum, regardless of estimated glomerular filtration rate (eGFR) status or structural kidney damage.

Inclusion criteria were as follows:

(1) confirmed diagnosis for at least 6 months;
(2) sufficient cognitive and communicative ability to articulate lived experiences;
(3) active disease management (e.g., outpatient visits, medication);
(4) willingness to participate in two or more in-depth interviews.

Recruitment was conducted at the pediatric nephrology outpatient department of C—University Hospital. Among 15 initially consented adolescents, two withdrew due to scheduling conflicts, resulting in a final sample of 13 (6 males, 7 females), with school levels ranging from middle school to university. The mean illness duration was 5 years and 7 months (range: 1–11 years). Nine participants had recurrent nephrotic syndrome (with 2–7 relapses), and four had other chronic glomerular diseases (one each: glomerulonephritis, nephritis, IgA nephropathy, and asymptomatic hematuria) (Table 1).

Data were collected between May and December 2015 through individual, unstructured, in-depth interviews. Each participant engaged in 2–4 interview sessions (60–90 min each), held in quiet, private hospital rooms selected to ensure comfort and confidentiality.

To minimize recall bias and social desirability bias, the researchers first established rapport through light, informal conversations about school, hobbies, or music before initiating the interviews. Interviews began with open-ended, non-leading prompts such as: “Can you describe what you’ve experienced while living with your kidney condition—at home, school, or with friends?” Follow-up questions were adapted based on emerging themes. Interviewers also observed non-verbal cues—tone of voice, posture, facial expression—to enrich contextual understanding.

All sessions were audio-recorded with consent, transcribed verbatim, and anonymized using coded identifiers. Data collection continued until no new concepts or themes emerged, confirming theoretical saturation.

**Table 1:** Participant Characteristics.

Participant	Sex	Age	Approximate Duration of the Disease	Diagnosis	Age at Diagnosis	Number of Recurrences	Medication Status
Participant 1	Male	14	5 years	Nephrotic syndrome	3rd grade	7 times	Currently taking medication
Participant 2	Female	13	2 years	Nephrotic syndrome	5th grade	4 times	Currently taking medication
Participant 3	Female	19	9 years	Nephrotic syndrome	4th grade	7 times	Currently taking medication
Participant 4	Female	19	8 years	Purpura Nephrotic syndrome	6th grade	2 times	Currently taking medication
Participant 5	Female	13	1 year	Purpura Nephrotic syndrome	5th grade	2 times	Currently taking medication
Participant 6	Male	16	5 years	Nephrotic syndrome	4th grade	6 times	Currently taking medication
Participant 7	Male	18	7 years	Nephrotic syndrome	4th grade	3 times	Not receiving medication, regular outpatient follow-up
Participant 8	Male	17	6 years	Nephrotic syndrome	4th grade	3 times	Currently taking medication
Participant 9	Male	16	11 years	Nephrotic syndrome	5 years old	3 times	Currently taking medication
Participant 10	Female	14	2 years	Asymptomatic hematuria	5th grade	Attends regular outpatient appointments	Currently taking medication
Participant 11	Female	13	2 years	Nephritis	4th grade	Attends regular outpatient appointments	Currently taking medication
Participant 12	Male	19	3 years	Glomerulonephritis	10th grade	Attending regular outpatient appointments	Currently taking medication
Participant 13	Female	19	11 years	IgA nephropathy	1st grade	Attending regular outpatient appointments	Currently taking medication

Data for this study were collected between May and December 2015 through individual, unstructured, in-depth interviews. Each interview took place in a quiet and comfortable environment chosen by the participants, such as private consultation rooms or designated spaces within the hospital, to ensure psychological comfort and privacy. To establish rapport and minimize participant anxiety, the researchers began each session with light, informal conversation about everyday topics (e.g., school life, music, or hobbies) before moving into research-related questions. Each participant took part in two to four interview sessions, lasting approximately 60 to 90 min per session. The interviews employed open-ended prompts, beginning with questions such as: “Can you describe what you have felt or experienced while living with your kidney condition and managing your health at home, at school, and in social situations?” Follow-up questions were guided by emerging themes identified from earlier sessions. Throughout the process, the interviewer observed non-verbal cues, such as tone of voice, body language, and facial expressions, to enhance contextual understanding. Interviews were recorded with participants’ consent and transcribed verbatim immediately afterward. All identifiable information was removed and replaced with coded ID numbers to ensure anonymity. Data collection continued until no new concepts or themes emerged, confirming theoretical saturation.

### 2.4. Data Analysis

The research team worked collaboratively to analyze the interview data. During this process, any viewpoints that emerged were examined, contrasted with the raw data, and clarified with participants when required. The data analysis followed the approach outlined by Colaizzi [11]. First, the interview data were read multiple times to understand the essential experiences of children living with chronic kidney disease, with the goal of interpreting the emotions expressed by participants and attaining a comprehensive grasp of the data. Key phrases or sentences related to participants’ accounts of their disease experiences were underlined to identify significant statements. Subsequently, we examined the context of these statements, identified their significance, and rephrased them independently. Similarities among the reworded meanings allowed us to group items together and develop overarching themes, which were then synthesized into more abstract theme clusters. To ensure that the theme clusters accurately represented the spectrum of disease experiences observed, participants were consulted to verify their alignment with personal experiences.

### 2.5. Ensuring Rigor in the Research

Rigor was ensured through Guba and Lincoln’s trustworthiness criteria: credibility, transferability, dependability, and confirmability [12].

• Credibility: Researchers maintained prolonged engagement, conducted multiple interviews, and performed member-checking.
• Transferability: Participants were adolescents with ≥6 months disease history, ensuring relevant experiential data. Data collection continued until saturation.
• Dependability: A consistent coding process was developed and reviewed by an external panel (2 qualitative nursing scholars, 1 psychologist, 1 humanities expert).
• Confirmability: Bracketing was used to minimize researcher bias. Reflexive journals documented thought processes and emotional responses throughout the study.

All researchers had clinical experience in pediatric nephrology or child health nursing and were trained in qualitative methodology, enabling both empathic sensitivity and methodological rigor.

## 3. Results

### 3.1. Theme Clusters

From the raw data, we identified 176 significant phrases or sentences. Phrases with related meanings were grouped, resulting in 65 reconstructed meanings. Following the exclusion of participant-specific experiences and the organization of common meanings, 19 themes were identified, which were integrated into seven theme clusters (Table 2).

#### 3.1.1. Theme Cluster 1: Appearance and Worsening of Kidney Disease Symptoms

Participants noticed the onset of kidney disease symptoms, which then progressed. Initial hospital visits occurred when symptoms such as edema, weight gain, hematuria, or proteinuria were detected, often by family members or close acquaintances during school urine tests, leading to a subsequent diagnosis. Since nephrotic syndrome often appears between age 5 and 5th grade, patients are commonly at a developmental stage where disease recognition is limited. Because the disease does not resolve quickly, patients are required to adhere to long-term medication regimens, dietary management, and activity restrictions. Furthermore, stress, colds, minor illnesses, and overexertion served as exacerbating factors.

Theme 1: Appearance of Kidney Disease Symptoms

“They detected blood in my urine during a school screening. It couldn’t be seen with the naked eye.”

(Participants 10, 11)

“My abdomen and face became swollen, … I assumed it was weight gain, but [following someone’s suggestion] I underwent tests at the hospital, and they found protein in my urine.”

(Participant 2)

Theme 2: Kidney Disease Symptoms Becoming More Prominent

“I tend to bottle things up until I reach my breaking point, so I sometimes experience multiple sources of stress simultaneously. I may have difficulties with other students at school … or my parents can upset me early in the morning. At times, they reprimand me quite sternly. Then my older sister, trouble at school, plus my evening part-time job with a challenging customer … when all of this happens while I’m already in a negative mood, it pushes me to my limits.”

(Participant 12)

#### 3.1.2. Theme Cluster 2: Restrictions in Daily Living

From the point of diagnosis, adolescents with chronic kidney disease must manage their health challenges while trying to maintain normal physical activities and daily routines at home and school. This management often involves adhering to specialized diets, limiting strenuous activity, and taking long-term medications. These treatment-related restrictions led participants to feel constrained in their everyday lives.

*Theme 3: Difficulties Controlling Diet*

“I can’t eat the same foods as my friends, and I have to regulate my intake. Because I can’t eat salty or spicy foods… I was unable to eat school meals, and I couldn’t have instant food, … Back in high school, I used to join my friends for meals during breaks. Now, I can’t even do that.”

(Participant 12)

“Food became a source of stress for me. When I dined with my friends, I had to eat something different, … I had to restrain myself [from eating inappropriate foods], which proved to be challenging.”

(Participant 6)

Theme 4: Difficulties with Physical Activity

“I really enjoyed playing soccer, and I was skilled at it. After starting high school, I would play soccer during lunchtime and dinnertime instead of eating, and that caused me significant stress.”

(Participant 5)

#### 3.1.3. Theme Cluster 3: Unstable Self-Control

The participants expressed anger about their persistent symptoms, the experience of becoming ill, and being hospitalized when they ate the same foods as their peers. Some resented their parents for “giving them this disease” and experienced feelings of despair. Frequent recurrences resulted in anxiety and depression, fear that their condition would not improve, uncertainty regarding treatment duration, and apprehension about their future, all of which increased their fear of the illness. At times, they blamed themselves and felt anxious when unable to manage their daily routines. The mix of despair, fear, anxiety, and depression contributed to uncertainty about their ability to achieve self-control during the process of gaining independence.

Theme 5: Despair

“It’s like, ‘Why am I like this? …’ … I wonder, ‘Why is it just me who is unwell? Why does it have to be me?’ I kept experiencing this persistent despair, but… I went to the hospital and was fine for a week. … I start to think I might recover, but then I’m discharged, and I… return to how I was, and I think, ‘Will I ever live like other kids again?’”

(Participant 7)

Theme 6: Fear

“I searched online, and it looked like the medicine doesn’t help [crying]. Recently, I’ve been starting to feel better when I take my medication, so I’m trying to take it routinely. I was really afraid. I didn’t speak to anyone, and sometimes when I feel down, I worry by myself since there’s no one I can consult, and even my mum and dad are not very knowledgeable.”

(Participant 8)

Theme 7: Tension

“You always have to choose bland food. You always need to be cautious. You have to be mindful about your choices whenever you eat.”

(Participant 2)

Theme 8: Depression

“Now, I no longer feel embarrassed, but at that time I did. Because all my friends were healthy, I felt unable to confide in anyone. I worry that my condition might worsen in the future, and I don’t want others to notice it, but whenever I return from the hospital, I experience a sense of depression. Why did I have to develop this disease [crying]?”

(Participant 3)

#### 3.1.4. Theme Cluster 4: Changes in Relationships with Friends

During a developmental period when peer acceptance is crucial, participants underwent notable changes in their friendships as a result of frequent outpatient appointments, hospitalizations, and the need to avoid excessive physical activity. These restrictions often led to withdrawal from friend groups. Participants reported feelings of isolation, particularly when excluded from conversations about shared events they missed due to medical appointments; this increased their sense of distance and, when they eventually rejoined their social circles, heightened feelings of being different and alienated.

*Theme 9: Sense of Isolation*

“I was admitted to the hospital early in the school semester, but after I was discharged, [my friends] would joke around about things that happened when I wasn’t there. … I felt left out.”

(Participant 2)

Theme 10. Sense of Alienation

“Initially, I felt stuck in between. Spending time with the other kids made the situation uncomfortable for everyone. Since my friends are aware of my illness, when they order food—something they know I used to enjoy—but see I’m not eating, they can tell something is wrong. This mutual discomfort led me to stop joining them altogether. … At first, it was manageable, but as it persisted, our closeness diminished.”

(Participant 7)

#### 3.1.5. Theme Cluster 5: Sensitivity About a Decrease in Achievements Due to Disease

Due to imposed activity restrictions and awareness of physical limitations during treatment, participants became increasingly aware of the disease’s impact on their future prospects. This led to inner conflict about whether to abandon their aspirations or modify their career goals. Ongoing outpatient visits and frequent hospitalizations resulted in missed classes, declining academic performance, and a sense of academic stagnation. Participants also reported diminished motivation to achieve as they were unable to engage in the activities considered typical for their healthy peers. The heightened sensitivity to decreasing achievements was particularly impactful during adolescence, a formative stage for developing self-identity, making career decisions, and preparing for future employment.

Theme 11: Awareness of the Disease’s Effects on Planned Career Paths

“I aspire to become an air steward. Health management is a critical aspect for someone in this profession. It is essential to maintain physical fitness. The job requires standing for extended periods, serving customers, and lifting heavy objects, which places significant strain on my back. Overexertion sometimes leads to recurrence of my symptoms. I am concerned that these health issues could prevent me from achieving my aspirations. I have devoted myself to this goal, and the thought of not being able to pursue it is deeply unsettling. That possibility is what frightens me the most.”

(Participant 3)

Theme 12: Treatment Process Interfering with Learning

“When I was first diagnosed with anemia, I found myself unable to participate in many activities I enjoyed, and my capacity to study was limited. If I try to focus intently on something, I experience dizziness, so I spend most weekends resting at home. While studying, I am unable to remain seated for long periods, and I frequently need to adjust my posture, which becomes another source of stress.”

(Participant 12)

Theme 13: Decreased Desire for Achievement

“Since I have glomerulonephritis, I feel I am at a disadvantage from the outset. I cannot help but think that I might perform better if I did not have this disease, although I realize it might sound like I am making excuses.”

(Participant 12)

“I dislike the fact that I am unable to do what others can. I wish I could live as others do. For example, I simply want to eat instant noodles like other young people.”

(Participant 3)

#### 3.1.6. Theme Cluster 6: Efforts to Maintain a Normal Daily Life

Among the participants, all but one developed their disease at age five and received their diagnosis during elementary school. Initially, most participants did not take their diagnosis seriously; however, as they matured, recurrent episodes, worsening symptoms, and experiences—either direct or vicarious—increased their awareness of the condition’s seriousness. As they identified circumstances that exacerbated their symptoms, they proactively began to seek information about their illness. They made deliberate choices to limit certain behaviors, which altered how they perceived their situation. When their symptoms deteriorated after overexerting themselves or disregarding dietary restrictions, they acknowledged their limitations and accepted, “I can’t do this anymore.” Through a process of experimentation, participants determined the extent of self-control necessary and achieved a functional balance.

Over time, the majority of participants adjusted to the changes imposed by their illness and incorporated management strategies into their daily routines. They implemented dietary restrictions, committed to maintaining a low-salt diet, and improved their eating habits, such as by bringing packed lunches to school or avoiding eating out. To increase adherence to medication, some set reminders to ensure timely dosing. Social interactions were modified accordingly; they either decreased the number of meetings with friends or adapted these occasions as needed. These efforts enabled participants to better comply with their regimen and facilitated their adjustment to living with a chronic illness.

Theme 14: Re-evaluating their Situation

“I’ve experienced frequent relapses since 4th grade. Initially, they told me that such recurrence was typical for this disease and that it would improve with adulthood. However, when I relapsed again as a college student, I questioned, ‘Wasn’t it supposed to improve after becoming an adult? Why am I still having relapses?’ They then explained that repeated relapses diminish the likelihood of improvement.”

(Participant 3)

Theme 15: Practical Compromise

“During the first six months, my life was almost entirely centered around managing my disease. After school, I would return home and was unable to eat freely, particularly having to avoid wheat-based foods. After about a year of following this, the continuous restriction became extremely stressful. When I chose to accompany my friends and eat as they did, I noticed a decline in my physical condition. Now, I believe I have managed to find a more balanced approach.”

(Participant 7)

Theme 16: Compliance with Treatment

“I check the sodium content and select foods that are low in sodium. Sweet foods usually contain less sodium. I either eat [*Satto-bap*] or opt for sweet chocolate snacks, as they also have minimal salt.”

(Participant 4)

“I avoided instant foods and brought a packed lunch to school. For both lunch and dinner, I made a conscious effort to have my mum prepare meals for me.”

(Participant 8)

#### 3.1.7. Theme Cluster 7: Psychological, Physical, and Social Strengthening

As participants reduced their social activities, they spent more time reflecting and gained a deeper appreciation of their health. They became increasingly attentive to their physical well-being and started contemplating their personal lives and future. Coping with illness fostered gratitude for their parents’ support and motivated them to reciprocate. During physical education classes, while remaining in the classroom, they engaged with peers who differed from themselves and developed a better understanding of others. This experience helped them become more empathetic and accepting, even when their views diverged from those of their friends. Their frequent hospital experiences enhanced their social skills and fostered independence. Consequently, by managing their disease over time, participants demonstrated substantial psychological development. They assumed greater responsibility in their disease management. Recognizing the value of health and the role of self-management in preventing recurrence, many proactively sought information about their illness. Medication adherence became an established aspect of daily life, and they monitored side effects independently. Sustained dietary management led to modified eating behaviors, and they actively rejected harmful behaviors like alcohol and tobacco consumption. Instead of viewing their condition with negativity, participants transformed it into motivation: Their illness inspired them to study more diligently and adopt lifestyles aligned with their circumstances.

Theme 17: Internal Growth

“I feel that this experience offered me a chance to become more mature. I caused significant emotional and financial difficulties for my parents. Initially, when I experienced a relapse, my immune system became very weak, necessitating a two-person room. It was extremely costly, and since we did not have insurance, financial challenges arose. My parents expressed concerns such as, ‘How can your kidneys already be impaired at your age? What if you need a transplant in the future?’ These situations made me realize that I needed to become a better son. If I had not been ill, I likely would not have reflected on these issues.”

(Participant 9)

Theme 18: Physical Strengthening

“I pay greater attention to my health now because I value being physically active. In middle school I played baseball, and in high school, I participated in soccer. I frequently sustained injuries, including ligament tears and swelling, but I now try to protect my body more. Since focusing on taking care of myself, I avoid being overly aggressive when playing sports. I believe I have become more even-tempered now.”

(Participant 5)

Theme 19: Social Strengthening

“I strive to avoid having specific expectations about outcomes, and I do not dwell on negative thoughts, choosing instead to live in the present with the belief that things will ultimately resolve.”

(Participant 10)

### 3.2. General Structure of the Disease Experiences of Children with Chronic Kidney Disease

This study identified the essential structure underlying the disease experiences of children with chronic kidney disease. In contrast to their healthy peers, these children encountered a variety of symptoms and daily life restrictions. Participants exhibited unstable self-regulation, with feelings of frustration, fear, tension, depression, and social isolation resulting from their illness. Their illness affected their career aspirations, created obstacles to their education, and contributed to reduced motivation for achievement during treatment.

**Figure 1:**
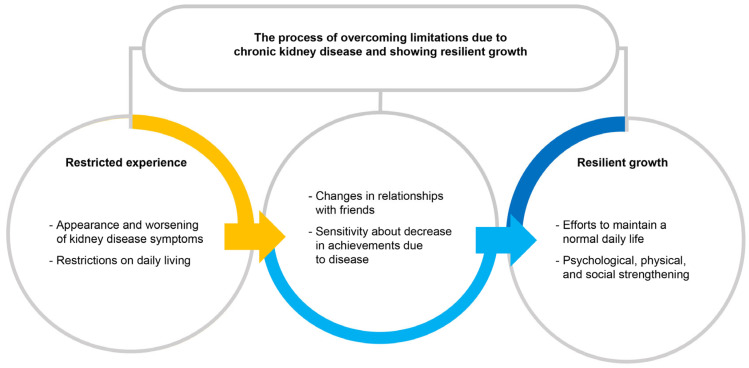
The process of adapting to the challenges posed by chronic kidney disease and demonstrating resilient growth.

Over time, however, these children began to transcend the limitations imposed by their condition. As they came to terms with their circumstances and adopted practical adjustments, they became more receptive to treatment regimens and made conscious efforts to lead a typical daily life. These adjustments facilitated internal development, expanding their self-awareness and empathic understanding toward others. The children also achieved physical strengthening through adopting healthier habits and developed social resilience by preparing for future opportunities that fit their specific context. The core of the disease experience for children with chronic kidney disease is maintaining a healthy day-to-day life amid illness, and is characterized as a “process of elastic growth through overcoming the limitations caused by chronic kidney disease” (Figure 1).

## 4. Discussion

This phenomenological study explored the lived experiences of adolescents diagnosed with chronic or recurrent glomerular diseases. Through qualitative analysis, seven thematic clusters were identified, illustrating how these individuals perceived and adapted to the complex realities of living with a chronic renal condition. The core theme that emerged was characterized as “overcoming limitations due to chronic illness and demonstrating resilient growth.”

Participants reported that the onset and recurrence of symptoms disrupted their daily lives and contributed to emotional challenges. These difficulties were especially pronounced during adolescence, a period marked by identity development, autonomy, and social integration. Participants described frequent experiences of anxiety, frustration, and social withdrawal—findings consistent with previous research highlighting the psychosocial burden in adolescents with chronic illnesses [13,14].

Peer relationships were notably affected. Some participants described distancing themselves from friends due to school absences, dietary restrictions, or fear of stigma, echoing meta-analytic findings that chronic illness can impair social functioning in youth [15]. In parallel, academic difficulties emerged as a major concern. Participants reported missed classes, reduced concentration, and altered future aspirations, reinforcing earlier literature on the educational impact of pediatric chronic disease [16,17,18].

Despite these challenges, many adolescents demonstrated growth and resilience. Through repeated cycles of illness and recovery, they developed practical self-care strategies, treatment adherence routines, and emotional insight. These findings underscore the need for structured psychosocial support that fosters self-management, emotional regulation, and long-term adjustment [19,20].

Although the study centered on adolescents’ voices, the role of caregivers cannot be overlooked. Several participants described parental involvement in medication management, emotional reassurance, and hospital coordination. However, caregiver perspectives were not directly included. Future research should incorporate parental viewpoints to better understand the interplay between adolescent autonomy and family support systems.

From a multidisciplinary perspective, the findings emphasize the need for integrative care involving nephrologists, pediatricians, nurses, psychologists, and school-based professionals. Adolescents living with chronic glomerular conditions frequently navigate interconnected medical, emotional, and social challenges. Therefore, holistic care models that merge clinical management with psychological and educational interventions may enhance adherence, mental well-being, and long-term quality of life. This aligns with contemporary pediatric nephrology guidelines advocating patient-centered, team-based approaches.

Additionally, although this study focused on psychological and social experiences, participants often described symptom flares associated with emotional distress or inconsistent medication use. While these associations were not quantitatively tested, they raise important clinical questions. Future mixed-method research should explore how psychological variables—such as anxiety, depression, or coping styles—relate to clinical outcomes including relapse frequency, complications, or treatment nonadherence.

All participants were between 14 and 19 years of age, a developmental window that spans middle to late adolescence. Although the study did not conduct age-based comparisons, participants’ narratives suggest developmental differences in emotional maturity, disease understanding, and coping strategies. These observations are visually summarized in Table 3, which presents a developmental overview of how illness experience and self-regulation evolved with age. Future studies should further investigate these differences using age-stratified designs to tailor age-appropriate interventions and transition planning.

This study has several limitations. First, it was conducted at a single institution with a small sample size (n = 13), which may limit generalizability. Second, the diagnostic composition was skewed toward recurrent nephrotic syndrome, reducing diversity across the spectrum of chronic glomerular diseases. Third, the data were collected in 2015; since then, advances in pediatric nephrology care, psychosocial services, and school reintegration programs may have changed the landscape of adolescent illness experience.

Furthermore, the study did not include objective clinical data such as disease stage, treatment modality, or presence of complications. This omission limits the ability to contextualize the psychological findings in relation to medical severity. It also did not assess how emotional responses (e.g., anxiety, depressive symptoms) may correlate with clinical trajectories (e.g., relapse rates, hospitalizations). Future mixed-method studies should address these relationships to provide a more integrated understanding of adolescent adaptation. Lastly, caregiver perspectives were not included, despite their indirect influence on participants’ illness management and emotional regulation.

These limitations point to important directions for future research. Longitudinal, multi-site studies incorporating clinical data, caregiver input, and age-based subgroup analyses are warranted. Such designs could improve the precision of psychosocial interventions and inform policy on adolescent-centered chronic care strategies.

## 5. Conclusions

Employing a phenomenological methodology, this study sought to clarify the lived experiences of adolescents diagnosed with chronic or recurrent glomerular disease, including nephrotic syndrome, glomerulonephritis, and related conditions requiring long-term management. Through in-depth interviews, seven thematic clusters were identified: “appearance and worsening of symptoms,” “restrictions in daily living,” “unstable self-control,” “changes in peer relationships,” “sensitivity about a decrease in achievements,” “efforts to maintain normalcy,” and “psychological, physical, and social strengthening.” The core essence of their experiences was defined as “overcoming limitations caused by chronic illness and demonstrating resilient growth.” Despite facing substantial emotional and social challenges, participants showed a capacity for adaptation, reflection, and behavioral change over time. These findings provide valuable insights into the psychosocial dynamics of adolescent chronic illness and offer foundational data for the development of individualized, age-sensitive, and interdisciplinary care strategies.

However, the study’s findings should be interpreted with consideration of certain contextual limitations. The sample was drawn from a single medical institution, included a small number of participants (n = 13), and predominantly comprised individuals with recurrent nephrotic syndrome. Moreover, the data were collected in 2015, and subsequent changes in clinical care, social environments, and school-based support may influence the present-day applicability of the findings.

Future research should aim to address these limitations by adopting multi-center and longitudinal designs with more diverse diagnostic representation. It is also recommended to develop validated assessment tools that can capture the subjective dimensions of adolescents’ experiences with chronic glomerular disease. Additionally, a conceptual framework of resilient growth specific to this population should be established to guide targeted interventions in both clinical and community settings.

## Figures and Tables

**Table 2:** Disease experience of children with chronic glomerular disease.

Theme Clusters	Theme	Formulated Meaning
Appearance and worsening of kidney disease symptoms	Appearance of kidney disease symptoms	• Swelling • Urine changes (hematuria, proteinuria) • Skin changes
	Symptoms becoming more prominent	• Symptoms worsening after catching a cold • Symptoms worsening after experiencing heavy stress • Symptoms worsening after lack of dietary control or not taking medication • Recurrence after excessive activity
Restrictions on daily living	Difficulty with dietary control	• Difficulties because of desire for food • Discomfort due to having to eat differently from peers
	Difficulties with physical activity	• Sense of resistance to exercise restrictions • Desire to exercise • Concerns about declining physical fitness
Unstable self-control	Despair	• Fluctuating disease symptoms • Dissatisfaction at differences with friends • Resentment toward parents
	Fear	• Anxiety about repeated recurrence • Concern that they will not get better • Uncertain treatment duration
	Tension	• Thinking that they always have to be careful • Always suppressing themselves • Feeling guilt about failures of control
	Depression	• Sadness about uncertain prognosis • Becoming sadder due to increasing disease awareness
Changing relationships with friends	Sense of isolation	• On the outskirts of peer groups • Leaving previous friend groups
	Sense of alienation	• Becoming distant from friends • Feeling different when socializing with friends
Sensitivity about a decrease in achievements due to disease	Awareness of the effects of disease on career path	• Considering a change of career path • Anxiety about potentially not being able to find an occupation • Fear of missing out on their dreams
	Treatment process interfering with learning	• Difficulty focusing on schoolwork • Academic stagnation due to hospitalization and hospital visits for treatment • Stress due to difficulties keeping up with classes
	Decreased desire for achievement	• Sense of inferiority due to disease • Loss of motivation to pursue possibilities • Difficulty concentrating
Efforts to maintain a normal daily life	Re-evaluating their situation	• Increased understanding of their disease through treatment • Seeking information • Setting intentional limits
	Practical compromise	• Acknowledgment • Finding a middle ground • Identifying foods that must not be eaten • Activities that do not cause excessive physical burden
	Compliance with treatment methods	• Implementing measures for a low-salt diet • Pursuing a healthy diet • Taking medication on time
Psychological, physical, and social strengthening	Internal growth	• Self-reflection • Maturation • Becoming more understanding • Developing independence and sociability
	Physical strengthening	• Taking time for themselves • Recognizing the importance of their bodies • Managing factors that could cause recurrence • Actively managing medications • Actively learning about their disease • Healthy lifestyle habits that benefit their bodies • Rejection of alcohol and tobacco
	Social strengthening	• Leading a life that is faithful to the present moment • Studying even harder • Maintaining normal friendships • Preparing a future suited to their circumstances

**Table 3:** Developmental Progression of Disease Perception and Coping among Adolescents with Chronic Glomerular Disease.

Developmental Stage	Disease Understanding	Emotional Response	Coping Strategies
Early Adolescence (~13–14 yrs)	Limited understanding; depends on parents for explanation	Confusion, fear, embarrassment	Passive compliance; avoids discussion of illness
Middle Adolescence (15–16 yrs)	Begins to recognize chronic nature of disease	Anxiety about relapse, comparison with peers	Attempts self-regulation, seeks peer acceptance
Late Adolescence (17–19 yrs)	Deeper understanding of long-term impact and self-responsibility	Frustration, acceptance, planning for future	Active self-management; prepares for adult care

Note: Based on thematic analysis. Not statistically validated.

## Data Availability

The original contributions presented in this study are included in the article. Further inquiries can be directed to the corresponding author.
